# Adolescent binge drinking in the West of Ireland: associated risk and protective factors

**DOI:** 10.1186/s12889-023-15577-z

**Published:** 2023-06-05

**Authors:** Ciara Kelly, Emmet Major, Michéal Durcan, Diarmuid O’Donovan, Áine McNamara

**Affiliations:** 1 PricewaterhouseCoopers Ireland, 1 North Wall Quay, North Dock, Dublin 1, Ireland; 2 Department of Public Health West, Health Service Executive, Merlin Park, Galway, Ireland; 3 Western Region Drug & Alcohol Task Force, Galway Roscommon Education Training Board, Parkmore, Galway, Ireland; 4 Western Region Drug & Alcohol Task Force, Health Service Executive, Parkmore, Galway, Ireland; 5 School of Medicine, Dentistry and Biomedical Sciences, Centre for Public Health, Queen’s University, Belfast, BT97BL UK

**Keywords:** Adolescent, Alcohol, Binge drinking, Risk factors, Protective factors

## Abstract

**Background:**

Alcohol is a leading cause of morbidity and mortality worldwide. Adolescence is when alcohol use typically begins. Harmful patterns of alcohol consumption, such as binge drinking, may emerge during adolescence and become established. This study aimed to examine potential risk and protective factors for binge drinking among 15–16-year-old adolescents in the West of Ireland.

**Methods:**

This was a cross-sectional secondary analysis of 4473 participants from the Planet Youth 2020 Survey. The outcome was ever binge drinking, defined as ever consumption of five or more drinks in a two-hour period or less. Independent variables were selected a priori following review of peer-reviewed literature and were grouped as individual, parents and family, peer group, school, leisure time and local community factors. Statistical analysis was completed using SPSS version 27. Differences in medians and means for continuous variables were examined using the Mann–Whitney U test and Independent Samples t-test respectively. Multivariable logistic regression was used to examine independent associations between potential risk and protective factors and ever binge drinking. A *p*-value of < 0.05 was deemed statistically significant.

**Results:**

The prevalence of ever binge drinking was 34.1%. Self-rated ‘bad/very bad’ mental health (adjusted Odds Ratio (aOR) 1.61, 95% CI 1.26–2.06, *p* < 0.001), current cigarette use (aOR 4.06, 95% CI 3.01–5.47, *p* < 0.001) and current cannabis use (aOR 2.79, 95% CI 1.80–4.31, *p* < 0.001) increased odds of ever binge drinking.

Parental supervision (aOR 0.80, 95% CI 0.73–0.88, *p* < 0.001) and negative parental reaction to adolescent drunkenness (aOR 0.51, 95% CI 0.42–0.61, *p* < 0.001) reduced odds of ever binge drinking. Getting alcohol from parents increased odds of ever binge drinking (aOR 1.79, 95% CI 1.42–2.25, *p* < 0.001).

Adolescents with friends who drink alcohol had almost five times higher odds of ever binge drinking (aOR 4.59, 95% CI 2.65–7.94, *p* < 0.001). Participating in team/club sports also increased odds of ever binge drinking (aOR 1.30, 95% CI 1.07–1.57, *p* = 0.008 for 1–4 times/week, aOR 1.52, 95% CI 1.07–2.16, *p* = 0.020 for ≥ 5 times/week).

**Conclusion:**

This study identifies individual and social environment factors associated with adolescent binge drinking in the West of Ireland. This can inform intersectoral action to protect adolescents from alcohol-related harm.

**Supplementary Information:**

The online version contains supplementary material available at 10.1186/s12889-023-15577-z.

## Background

Alcohol is a major public health issue. The volume of alcohol consumed, and the pattern of consumption are key factors in determining alcohol use-related harms [1]. The World Health Organization (WHO) considers harmful use of alcohol a causal factor in over 200 disease and injury conditions, whereby harmful use encompasses that which causes adverse health and social consequences to the drinker, those around the drinker and wider society, and patterns of use associated with detrimental health impacts [2, 3] The spectrum of adverse health consequences due to harmful alcohol use includes alcohol-related cancers, liver and cardiovascular disease, mental and behavioural disorders, and physical and psychological injuries due to alcohol-related violence and road traffic accidents [3]. In 2016, 5.3% of deaths and 5.1% of disability-adjusted life years (DALYs) globally were attributed to alcohol [3]. The non-health sectors impacts of alcohol-related harm, such as increased crime, economic losses and lost workforce productivity, are also significant [2].

Many factors influence the volume and pattern of alcohol consumption at a population level, from the level of the individual and community, to the broader sociocultural, legislative and policy context [3]. As a result, there are substantial geographic differences in alcohol consumption internationally [4]. In 2019, the average volume of alcohol consumed across all 38 Organisation for Economic Co-operation and Development (OECD) countries was 8.7 L per capita, ranging from 12.9 L in Lativa to 0.1 L in Indonesia [5]. Global changes in consumption trends over time were described in the 2018 WHO Global Status Report on Alcohol and Health [6]. While the WHO European Region has the highest per capita alcohol consumption, this has decreased over time, alongside stable consumption trends in the African Region, Americas and Eastern Mediterranean Regions, and increases in the Western Pacific and Southeast Asia Regions [2].

Patterns of alcohol consumption also vary by country. WHO considers heavy episodic drinking (HED)—defined as consumption of at least 60 g of pure alcohol at least once in the previous 30 days, and often referred to colloquially as binge drinking—the best indicator for alcohol consumption patterns associated with adverse health consequences [7]. While the overall prevalence of HED has decreased globally since 2000 (22.6% to 18.2% in 2016), in certain countries of Eastern Europe and Sub-Saharan Africa it remains high at over 60% of current drinkers [6]. In Europe, the 2016 prevalence of 30-day HED ranged from 62.3% in Lithuania to 28.3% in Ukraine [4].

In the Republic of Ireland (referred to as Ireland hereafter), nationally representative population-level surveys such as the annual Healthy Ireland Survey and National Drug and Alcohol Survey have identified a high prevalence of binge drinking (using a definition of consuming six or more standard drinks in a single drinking occasion) among the Irish population, with an overall declining trend over time [8, 9]. In the 2021 Healthy Ireland Survey, 22% of Irish drinkers aged 15 and older (15% of the population) reported binge drinking in a typical drinking occasion, a decrease compared to 37% of drinkers and 28% of the population respectively in 2018 [8]. The 2019–2020 National Drug and Alcohol Survey identified a more nuanced trend, with a decrease since 2002–2003 in monthly HED among 15–24 and 25–34 year olds (73.7% to 56.4%) accompanied by a stable and increased prevalence among 35–49 and 50–64 year olds respectively [9]. Sociodemographic differences in binge drinking have also been identified among the Irish population. The 2016 Healthy Ireland Survey found binge drinking to be more common among Irish men (35%) compared to women (10%), among younger age groups (31% of drinkers aged under 25) compared to older (13% of drinkers aged 65 or older), and among those living in disadvantaged areas (43%) compared to more affluent areas (33%) [8].

Adolescence, a period of biological, cognitive, emotional and social development, is when alcohol use typically begins [1, 10]. This is of significant concern, given adolescents are more vulnerable to the adverse health effects of alcohol consumption compared to adults. Harmful patterns of alcohol consumption, such as binge drinking, may emerge during adolescence and become established. This pattern of consumption places adolescents at greater risk of experiencing alcohol-related harm as well as other substance use in later life [10–12]. In the 2019 European School Survey Project on Alcohol and Other Drugs (ESPAD) Survey, one in three students (34%) from the 35 participating countries reported at least one episode of HED (defined as consumption of five or more drinks in a single occasion) in the previous 30 days [13]. Since 1995, 30-day HED prevalence as measured by ESPAD has increased among females (30% to 34%) and decreased for males (41% to 36%). Among Irish adolescents, there was a 32% 30-day prevalence of HED in ESPAD 2019 (33% for males and 32% for females) – a notable increase from 2015 (28%) and change from a previously declining trend since 2003. Understanding the determinants of binge drinking among adolescents is important to inform public health policy and intersectoral action to protect adolescents from alcohol-related harm. This knowledge can also inform comparisons between countries. A substantial body of international literature has examined possible determinants of adolescent binge drinking, generally termed ‘risk factors’ and ‘protective factors’. These factors are frequently grouped into specific domains—for example, factors at the sociodemographic level of the individual, as well as those specific to adolescents’ wider social environment, such as parental, peer, school and local community-related factors. There is heterogeneity in measurement of these exposure variables across the literature, and in definitions of binge drinking with respect to the volume of alcoholic drinks consumed and the time period in which consumption is measured.

While the body of international literature on risk and protective factors for binge drinking is sizeable, examination of risk and protective factors for binge drinking specifically among adolescents living in the Ireland is a notably understudied area in comparison to other high-income countries. Therefore, to further understanding of determinants of adolescent binge drinking in Ireland, this study aimed to examine the prevalence of, and potential risk and protective factors associated with, binge drinking among a cohort of 15–16-year-old adolescents in the West of Ireland who participated in the Planet Youth 2020 Survey.

## Methods

### Study design

This study is a cross-sectional secondary analysis of data from the second Planet Youth Survey in the West of Ireland (conducted in December 2020), based on the international Planet Youth Survey [14].

### Study population

The target population of the Planet Youth 2020 Survey was 15–16-year-old students from all post-primary schools and Youthreach (the Irish government education, training and work programme for early school leavers) centres in the three counties of Galway, Mayo and Roscommon (*n* = 6373) in the West of Ireland [15]. For the 2020 Survey, all post-primary schools and Youthreach centres in each of the three counties were invited to take part, and all agreed to participate (81 schools and ten Youthreach centres). Students and their parents (and/or legal guardians) were provided with information regarding the Planet Youth Survey, and were given the option to not participate if they wished.

In total, 5083 completed surveys were returned from the target population of 6373 (response rate 79.8%). Following data cleaning and validation, 5000 surveys remained (adjusted response rate 78.5%). For the present study, participants aged older than 15–16-years-old were excluded (*n* = 527), giving a study population of 4473 adolescents (70.2% of the target population).

### Data extraction

In the Planet Youth 2020 Survey, data were collected using the Planet Youth Survey questionnaire, a standardised instrument used in each country which has adopted the Planet Youth process. For the present study, a data request form was submitted to the Department of Public Health (Health Service Executive) West, the data holder at the time of this study, who provided the anonymised dataset in Statistical Package for the Social Sciences (SPSS) format. Data were anonymised for the Planet Youth 2020 Survey through removal of the school identifier for participants, and collapsing of certain variable categories. The relevant dependent and independent variables for this study were extracted from the Planet Youth 2020 Survey data. A data dictionary is provided as an additional file (see Additional file 1), which details all variables extracted and recoding completed for the present study.

### Variables

#### Dependent variable

The dependent variable, self-reported history of ever binge drinking, was defined as ever consumption of five or more drinks in a two-hour period or less. In the Planet Youth 2020 Survey, participants were asked ‘This is a question about the amount of alcoholic drinks (e.g. beer, wine, spirits, shots) that you might drink at one time. How often have you had?’ Participants were asked to respond for the number of times they had consumed five or more alcoholic drinks in a two-hour period or less, from categories of ‘Never’, ‘1–2 times’, ‘3–5 times’, ‘6–9 times’, ‘10–19 times’, ‘20–39 times’ or’40 times or more’. This was recoded as a binary variable, with participants categorised as having ‘Never’ binge drank, or ‘Ever’ binge drank if they reported having done so one or more times. Selection of this outcome definition was informed by a review of peer-reviewed literature on risk and protective factors for adolescent binge drinking, and of definitions of binge drinking from selected high-income countries and public health organisations [16–21]. The decision to re-categorise binge drinking as a binary outcome was also informed by the international literature, and examination of outcome measures in the included studies. Across the literature, acknowledging some heterogeneity, binge drinking was most frequently defined as consumption of five or more drinks in a single drinking occasion, using a binary categorisation (i.e. a yes or no response) [22–45]. However, the time frame for reporting binge drinking episodes varied substantially across the included studies (e.g. past 30-day versus past 12-months versus lifetime episodes of binge drinking).

#### Independent variables

Selection of independent variables was informed by review of peer-reviewed literature and the theoretical background to the Planet Youth process. These variables were grouped according to the Planet Youth process domains of the adolescent and their social environment, as follows:

- Sociodemographic factors: Gender, Ethnicity, Maternal education
- Individual factors: Self-rated mental health, Current cigarette use, Current cannabis use
- Parent/family factors: Parental supply of alcohol to adolescent, Parental drunkenness, Parental reaction to adolescent drunkenness, Parental supervision
- Peer group factors: Having friends that drink alcohol
- School factors: School engagement
- Leisure time and local community factors: Team or club (team/club) sport participation, Sourcing alcohol from friends, Sourcing alcohol from another (non-parent) adult

Two scale (continuous) variables were derived – parental supervision and school engagement. Parental supervision was derived from responses to the statements: ‘My parents/carers know who I am with in the evenings’ and ‘My parents/carers know where I am in the evenings’. Participants were asked how well these statements applied to them, with responses ranging from ‘Very Well’ to ‘Very Poorly’, corresponding to a score of 1–4. Responses were reverse coded and combined to create a single scale variable (range 2–8) (Cronbach’s Alpha 0.847), followed by scale standardisation, such that a one standard deviation increase on the scale (i.e. increasing parental supervision) is represented by the odds ratio. This scale is consistent with that used in previous research by the Icelandic Planet Youth group, and in previous Irish research on risk and protective factors for substance use [46–48].

School engagement was derived from responses to the statements ‘I find schoolwork pointless’, ‘I find schoolwork boring’, ‘I am poorly prepared for classes’ and ‘I feel I don’t put enough effort into my schoolwork’. Participants were asked how well these statements applied to them – responses ranged from ‘Almost Always’ to ‘Almost Never’, corresponding to a score of 1–5. Responses were combined to create a single scale variable (range 4–20) (Cronbach’s Alpha 0.754), followed by scale standardisation, such that a one standard deviation increase on the scale (i.e. increasing engagement with schoolwork/classes) is represented by the odds ratio. This construct of school engagement is consistent with that used in previous research by the Icelandic Planet Youth group [46].

All other independent variables were categorial.

### Statistical analysis

IBM SPSS version 27 was used for statistical analysis. Descriptive analyses were performed to examine the number of binge drinking episodes reported by participants (Table 1), and the overall prevalence of ever binge drinking among, and characteristics of, participants (Table2). The cross-tabulations procedure with Chi-Square tests was used to detect significant differences in proportions between categorical independent variables (Table 3). The Mann–Whitney U test and Independent Samples t-test were used for the parental supervision and school engagement variables respectively.

**Table 1:** Number of Times Participants Reported Ever Binge Drinking (*n* = 4390)

Number of Binge Drinking Episodes	*n*	(%) of all participants	(%) of ever binge drinkers
0 times	2893	65.9	N/A
1–2 times	615	14.0	41.1
3–5 times	337	7.7	22.5
6–9 times	196	4.5	13.1
10–19 times	186	4.2	12.4
20–39 times	79	1.8	5.3
40 times or more	84	1.9	5.6

*N/A* Not applicable

**Table 2:** Characteristics of the Study Population (*n* = 4473)

	**Valid**	
**Variables**	***n***	***n* (%)**
**Binge Drinking**	4390	
Ever		1497 (34.1)
Never		2983 (65.9)
**Sociodemographic**		
Gender	4395	
Male		2172 (49.4)
Female		2223 (50.6)
Ethnicity	4454	
White		4162 (93.4)
Non-White		292 (6.6)
Maternal Education	4429	
Tertiary		2320 (52.4)
Secondary		994 (22.4)
Primary		230 (5.2)
Didn’t Know		885 (20.0)
**Individual**		
Mental Health	4446	
Very good/good		2101 (47.3)
Okay		1447 (32.5)
Bad/very bad		898 (20.2)
Current cigarette use	4434	
Yes		560 (12.6)
No		3874 (87.4)
Current cannabis use	4395	
Yes		291 (6.6)
No		4104 (93.4)
**Parents and family**		
Parental supervision	4419	
Median (IQR)		7 (2)
Parental drunkenness	4432	
At least weekly		704 (15.9)
No/less than weekly		3728 (84.1)
Parental reaction to drunkenness	4399	
A bit against/wouldn’t care		1692 (38.5)
Totally against/against it		2707 (61.5)
Gets alcohol from parents	4396	
Never/rarely		3605 (82.0)
Sometimes/often/almost always		791 (18.0)
**Peer Group**		
Having friends that drink alcohol	4395	
Yes		3872 (88.1)
No		523 (11.9)
**School**		
School engagement	4353	
Mean (sd)		12.8 (2.9)
**Leisure Time/Source of Alcohol in Local Community**		
Team/club sports participation	4435	
Never		2014 (45.4)
1–4 times/week		2113 (47.6)
5 or more times/week		308 (7.0)
Gets alcohol from friends	4387	
Never/rarely		2928 (66.7)
Sometimes/often/almost always		1459 (33.3)
Gets alcohol from another adult	4392	
Never/rarely		3500 (79.7)
Sometimes/often/almost always		892 (20.3)

*IQR* Interquartile range, *sd* Standard deviation

**Table 3:** Comparison of Characteristics of the Study Population According to Binge Drinking Status (*n* = 3968)

	**Binge Drinking**		
	**Ever** ***n* (%)**	**Never** ***n* (%)**	
**Variables**	**1344 (33.9)**	**2624 (66.1)**	***p*-value**
**Sociodemographic**			
Gender			
Male	685 (51.0)	1251 (47.7)	
Female	659 (49.0)	1373 (52.3)	0.054
Ethnicity			
White	1291 (96.1)	2433 (92.7)	
Non-White	53 (3.9)	191 (7.3)	< 0.001
Maternal Education			
Tertiary	690 (51.3)	1420 (54.1)	
Secondary	342 (25.5)	572 (21.8)	
Primary	73 (5.4)	123 (4.7)	
Didn’t Know	239 (17.8)	509 (19.4)	0.034
**Individual**			
Mental Health			
Very good/good	539 (40.1)	1358 (51.7)	
Okay	439 (32.7)	855 (32.6)	
Bad/very bad	366 (27.2)	411 (15.7)	< 0.001
Current cigarette use			
Yes	385 (28.6)	95 (3.6)	
No	959 (71.4)	2529 (96.4)	< 0.001
Current cannabis use			
Yes	212 (15.8)	38 (1.4)	
No	1132 (84.2)	2586 (98.6)	< 0.001
**Parents and family**			
Parental supervision			
Median (IQR)	6 (2)	8 (2)	< 0.001
Parental drunkenness			
At least weekly	323 (24.0)	307 (11.7)	
No/less than weekly	1021 (76.0)	2317 (88.3)	< 0.001
Parental reaction to drunkenness			
A bit against/wouldn’t care	782 (58.2)	740 (28.2)	
Totally against/against it	562 (41.8)	1884 (71.8)	< 0.001
Gets alcohol from parents			
Never/rarely	957 (71.2)	2307 (87.9)	
Sometimes/often/almost always	387 (28.8)	317 (12.1)	< 0.001
**Peer Group**			
Having friends that drink alcohol			
Yes	1329 (98.9)	2172 (82.8)	
No	15 (1.1)	452 (17.2)	< 0.001
**School**			
School engagement			
Mean (sd)	13.3 (3.0)	12.6 (2.9)	< 0.001
**Leisure Time/Source of Alcohol in Local Community**			
Team/club sports participation			
Never	558 (41.5)	1219 (46.4)	
1–4 times/week	679 (50.5)	1235 (47.1)	
5 or more times/week	107 (8.0)	170 (6.5)	0.007
Gets alcohol from friends			
Never/rarely	459 (34.2)	2201 (83.9)	
Sometimes/often/almost always	885 (65.8)	423 (16.1)	< 0.001
Gets alcohol from another adult			
Never/rarely	725 (53.9)	2444 (93.1)	
Sometimes/often/almost always	619 (46.1)	180 (6.9)	< 0.001

*IQR* Interquartile range, *sd* Standard deviation

Univariable binary logistic regression analyses were performed for each independent variable (available as an additional file (see Additional file 2)). A multivariable logistic regression was then constructed to examine independent associations between independent variables and the outcome. Independent variables were sequentially added to the multivariable logistic regression according to their respective domain, beginning with Model 1 (available as an additional file (see Additional file 3)) containing the sociodemographic variables. Each subsequent model contained variables included in the previous model and the new variables to be added (available as an additional file (see Additional file 4)). Model 6 (Table 4) represents the main effects model with all independent variables included. Models were evaluated using the Nagelkerke (pseudo) R [2] measure, and the percentage accuracy in classification (PAC). Participants with missing data for any variables (*n* = 505) were excluded from the logistic regression to facilitate a complete case analysis.

**Table 4:** Analysis of Potential Risk and Protective Factors Associated with Ever Binge Drinking: Final Model (*n* = 3968)

	**Binge Drinking (Ever vs. Never)**		
**Variables**	**aOR**	**95% CI**	***p*-value**
**Sociodemographic**			
Gender			
Male	Ref		
Female	0.55	0.46–0.67	< 0.001
Ethnicity			
White	Ref		
Non-White	0.49	0.31–0.77	0.002
Maternal Education			
Tertiary	Ref		
Secondary	1.06	0.85–1.31	0.620
Primary	1.03	0.67–1.57	0.910
Didn’t Know	0.94	0.73–1.20	0.596
**Individual**			
Mental Health			
Very good/good	Ref		
Okay	0.90	0.73–1.11	0.313
Bad/very bad	1.61	1.26–2.06	< 0.001
Current cigarette use			
No	Ref		
Yes	4.06	3.01–5.47	< 0.001
Current cannabis use			
No	Ref		
Yes	2.79	1.80–4.31	< 0.001
**Parents and family**			
Parental supervision			
1 SD increase corresponds to	0.80	0.73–0.88	< 0.001
Parental drunkenness			
No/less than weekly	Ref		
At least weekly	1.25	0.98–1.59	0.071
Parental reaction to drunkenness			
A bit against/wouldn’t care	Ref		
Totally against/against it	0.51	0.42–0.61	< 0.001
Gets alcohol from parents			
Never/rarely	Ref		
Sometimes/often/almost always	1.79	1.42–2.25	< 0.001
**Peer Group**			
Having friends that drink alcohol			
No	Ref		
Yes	4.59	2.65–7.94	< 0.001
**School**			
School engagement			
1 SD increase corresponds to	1.26	1.15–1.38	< 0.001
**Leisure Time/Source of Alcohol in Local Community**			
Team/club sports participation			
Never	Ref		
1–4 times/week	1.30	1.07–1.57	0.008
5 or more times/week	1.52	1.07–2.16	0.020
Gets alcohol from friends			
Never/rarely	Ref		
Sometimes/often/almost always	5.98	4.95–7.21	< 0.001
Gets alcohol from another adult			
Never/rarely	Ref		
Sometimes/often/almost always	4.18	3.35–5.21	< 0.001

*aOR* Adjusted odds ratio, *95% CI* 95% Confidence interval, *Ref* Reference group, *SD* Standard deviation

Model Nagelkerke pseudo-r2 = 0.535, PAC = 83.2%

A *p*-value of < 0.05 was deemed statistically significant. Multicollinearity was tested for among independent variables, and was not considered a problem.

## Results

### Prevalence of ever binge drinking

Table 1 presents the number of times participants reported ever binge drinking. Approximately one-third (34.1%) of participants reported ever binge drinking (males 35.8%, females 32.5%). The majority of ever binge drinkers reported 1–2 and 3–5 episodes of binge drinking previously (41.1% and 22.5% respectively).

### Characteristics of participants

Table 2 presents the characteristics of participants. Table 3 presents a comparison of these characteristics according to binge drinking status.

There was no statistically significant difference in the proportion of males and females. The majority of participants were of White ethnicity (93.4%). The majority of participants were not current cigarette users (87.4%) or cannabis users (93.4%). Most participants (88.1%) reported having friends that drink alcohol. Approximately one-third (33.3%) and one-fifth (18.0%) reported they sometimes, often or almost always sourced alcohol from their friends and parents respectively. Significant differences in characteristics between ever versus never binge drinkers were observed for most independent variables.

### Results of the multivariable logistic regression

Results of the final model in the multivariable logistic regression (Model 6) are presented in Table 4.

### Sociodemographic factors

Compared to males, the odds of reporting ever binge drinking were almost 50% lower for females (aOR 0.55, 95% CI 0.46–0.67, *p* < 0.001). Participants of non-White ethnicity – compared to those of White ethnicity – had 51% lower odds of ever binge drinking (aOR 0.49, 95% CI 0.31–0.77, *p* = 0.002).

### Individual factors

Compared to adolescents reporting ‘very good/good’ mental health, the odds of ever binge drinking were 1.6 times higher (aOR 1.61, 95% CI 1.26–2.06, *p* < 0.001) for adolescents who reported ‘bad/very bad’ mental health.

Compared to non-current users, the odds of ever binge drinking among those that reported current cigarette use were 4 times higher (aOR 4.06, 95% CI 3.01–5.47, *p* < 0.001), while the odds for those that reported current cannabis use were almost three times higher (aOR 2.79, 95% CI 1.80–4.31, *p* < 0.001).

### Parent and family factors

For each one standard deviation increase in parental supervision score, the odds of ever binge drinking were 20% lower (aOR 0.80, 95% CI 0.73–0.88, *p* < 0.001). The odds of ever binge drinking were approximately 50% lower for participants who reported their parents would be against or totally against their becoming drunk (aOR 0.51, 95% CI 0.42–0.61, *p* < 0.001), compared to those whose parents would be a bit against it or would not care.

Adolescents who reported they sometimes, often or almost always get their alcohol from their parents had approximately 1.8 times higher odds of ever binge drinking (aOR 1.79, 95% CI 1.42–2.25, *p* < 0.001), compared to those who never or rarely did so.

Although parental drunkenness was significantly associated with adolescent binge drinking in the univariable analysis (OR 2.39, 95% CI 2.01–2.84, *p* < 0.001), this association became non-significant in the multivariable model (aOR 1.25, 95% CI 0.98–1.59, *p* = 0.071).

### Peer group factors

Compared to participants who stated none of their friends drink alcohol, the odds of ever binge drinking were almost five times higher for participants with any friends that drink alcohol (aOR 4.59, 95% CI 2.65–7.94, *p* < 0.001).

### School factors

For each one standard deviation increase in school engagement score (i.e. increasing engagement with schoolwork/classes), the odds of ever binge drinking increased (aOR 1.26, 95% CI 1.15–1.38, *p* < 0.001).

### Leisure time and source of alcohol in local community factors

Compared to participants who reported no team/club sport participation, the odds of ever binge drinking were 1.3 and 1.5 times higher for those who reported participating in team/club sports one to four times and five or more times per week respectively (aOR 1.30, 95% CI 1.07–1.57, *p* = 0.008 and aOR 1.52, 95% CI 1.07–2.16, *p* = 0.020).

Adolescents who reported they sometimes, often or almost always source alcohol from friends or a non-parent adult had approximately six and four times higher odds of reporting ever binge drinking (aOR 5.98, 95% CI 4.95–7.21, *p* < 0.001 and aOR 4.18, 95% CI 3.35–5.21, *p* < 0.001 respectively), compared to those who never or rarely did so.

## Discussion

### Prevalence of ever binge drinking

Just over one-third of the 4473 15–16-year-old adolescent participants in this study reported ever binge drinking, highlighting that this harmful pattern of underage alcohol use remains prevalent in Ireland. International variation in binge drinking definitions somewhat limits direct comparison between the prevalence of ever binge drinking observed in this study with previous international estimates. However, acknowledging this, some comparisons can be drawn. At regional level, in the 2018 Planet Youth Survey, 4,490 15–16-year-old adolescents in the West of Ireland were asked how often they had ever consumed five or more drinks in a one-hour period or less. Just under one-third (31.3%) reported ever doing so. It is therefore, highly concerning to note an increase since 2018 in the proportion of adolescents in the West of Ireland reporting consumption of large volumes of alcohol in very short spaces of time.

In considering possible reasons for this increase, it is important to note that the 2018 and 2020 Planet Youth Survey populations represent two sequential cohorts of adolescents aged 15–16-years-old. The main contextual difference at a societal level between these two waves of data collection and adolescent populations surveyed has been the COVID-19 pandemic. Throughout most of 2020 the Irish population, like many others worldwide, was subject to unprecedented societal restrictions to limit transmission of SARS-CoV-2, which substantially curtailed opportunities for social mixing [49, 50]. It is notable that the prevalence of ever binge drinking among participants remains high despite these restrictions being in place. One possibility is that in the absence of organised and supervised social events (particularly indoors) due to COVID-19 restrictions, some adolescents may have sought alternative unsupervised settings for socialisation, with greater potential for binge drinking behaviour.

At national and international level, the prevalence of binge drinking observed in this study is slightly higher than the 30-day HED prevalence identified in the 2019 ESPAD Survey for Ireland (32%), and similar to that observed across the 35 countries included (34%) [13]. This comparison is notably limited by the difference in outcome definition between ESPAD and this study with respect to the time period for consumption. Comparing with international literature which has examined the prevalence of ever binge drinking among adolescents using the same outcome definition as the present study, estimates vary by age range and nationality with an overall increasing prevalence with age, ranging from 0.6% among 10–11-year-old UK adolescents [32], 4% among adolescents in the USA aged 10–14-years-old [38], 10% among UK adolescents aged 14 [51], 23.1% among Brazilian adolescents aged 12 [27], to 27.4% among a cohort of German elite adolescent athletes (mean age 16.3 years old) [24].

### Risk and protective factors associated with ever binge drinking

Nine risk factors and four protective factors were independently associated with ever binge drinking among participants in this study. Female adolescents and adolescents of non-White ethnicity had reduced odds of ever binge drinking, compared to males and those of White ethnicity respectively. These findings are consistent with previous international studies [24, 30, 34, 38, 39, 42, 52, 53]. Substance use is generally more common in men [10], and while reasons for such differences in binge drinking by ethnicity are considered under-studied, suggestions in the literature include differences in cultural and traditional norms, as well as greater familial versus peer influence for non-White adolescents [42, 54]. No association was found in this study between maternal education as proxy for parental socioeconomic status (SES) and adolescent binge drinking. A 2016 systematic review of studies from 20 countries reported similarly inconclusive results, thought to be the result of heterogeneity in parental SES measurement [55].

Adolescents who self-rated their mental health as ‘bad/very bad’ had increased odds of ever binge drinking, supporting some previous cross-sectional studies that have identified greater odds of binge drinking among adolescents who self-reported experiencing depressive symptoms and suicidal thoughts [25, 37, 40]. However, longitudinal studies are needed to establish the true nature of the relationship between adolescent mental health and binge drinking, particularly given the potential for bi-directional associations [1].

Current cigarette and cannabis use were strongly associated with increased odds of ever binge drinking among participants, consistent with cross-sectional studies from other high-income countries. [35, 37, 42, 56], Such studies cannot, however, confirm the temporality of these relationships. It is known that risky substance use behaviours can co-occur among adolescents [40, 57], and the sequence of substance initiation and experimentation may vary between countries due to different sociocultural contexts [10].

Getting alcohol from parents increased odds of ever binge drinking among participants in this study, while parental supervision and negative parental reaction to adolescent drunkenness were identified as protective factors. These findings support a 2017 systematic review and meta-analysis which examined modifiable parenting factors associated with adolescent alcohol misuse [58]. Parental monitoring – considered synonymous with parental supervision – was reported as the strongest protective factor, while parental supply of alcohol to adolescents and favourable parental attitudes towards alcohol were identified as risk factors.

Unexpectedly, the positive association between parental drunkenness and adolescent binge drinking identified on univariable analysis did not retain significance in the final multivariable model. Previous studies have identified parental alcohol consumption and intoxication as risk factors for adolescent binge drinking [37, 45, 54, 58, 59]. Possible reasons for the lack of a significant association in this study include suboptimal statistical power to detect a significant association, the use of a binary categorisation for the study outcome, exposure measurement, and/or greater influence on participants of peer (versus parental) alcohol use.

It is known that adolescents are increasingly subject to peer influences in the transition between childhood and adulthood [60]. The majority of participants in this study reported having friends who drink alcohol, which was strongly associated with increased odds of ever binge drinking. This is consistent with identification of peer alcohol use as one of the strongest risk factors for adolescent binge drinking in previous studies [29, 35, 38, 56, 60–63].

A further unexpected finding was the positive association between adolescent school engagement and ever binge drinking. Previous studies have identified higher academic achievement as protective against adolescent binge drinking, with school truancy considered a risk factor [31, 34, 35, 40, 47, 56, 63]. The relationship observed in this study may be due to residual confounding due to factors not measured, given adolescents’ school experience may be influenced by many factors besides academic engagement, such as peer relationships, perceived support from school staff and non-academic extra-curricular activities.

With respect to the positive association observed between ever binge drinking and team/club sports participation, although supportive of some previous research [40, 64], it remains unclear whether sports participation and/or exercise itself influence adolescent alcohol consumption independent of the peer influences, opportunities for socialisation and alcohol-promoting cultures that may accompany sports team or club membership. This should be a priority for further exploration in future studies, particularly given physical activity is a positive lifestyle behaviour that should be encouraged among adolescents.

Approximately one-third (33.3%) and one-fifth (20.3%) of participants reported sourcing alcohol from their friends and another (non-parent) adult respectively. The positive associations observed between these alcohol sources and ever binge drinking are consistent with findings from previous studies [53, 65].

### Strengths and limitations

This study provides important insights on risk and protective factors for ever binge drinking among Irish adolescents, a relative evidence gap compared to the body of literature from other high-income countries. The most notable strength of this study is its large study population, and its use of secondary data from the Planet Youth 2020 Survey, a rich source of information on adolescent life in the West of Ireland. Response rates to the 2020 Survey were overall high, with relative consistency across the three counties surveyed, providing reassurance regarding the representativeness of the study population for the West of Ireland.^15^ The target population of the 2020 Survey included urban and rural populations, and as such, there is no reason to consider the population of this study to be markedly different to Irish adolescents nationally.

There are several limitations to this study. The cross-sectional study design precludes causal inferences and the establishment of temporal relationships between the exposures and outcome.

The binary categorisation of the study outcome (ever binge drinking) is a potential limitation of this study, as it precluded analysis of the relationship between each of the independent variables selected and the number of binge drinking episodes reported by participants. It is possible that the risk and protective associations identified may vary according to the number of times participants have binge drank previously. As this was not the aim of nor part of the analysis for this study, speculation on such differences cannot be made here. Additionally, as the Planet Youth questionnaire does not objectively quantify for participants what constitutes an ‘alcoholic drink’, it is also recognised that subjectivity is likely among participants responding to this question.

Information was not available regarding characteristics of those who opted not to participate in the Planet Youth 2020 Survey. Therefore, selection bias is possible among the study population. Recall bias is also possible, as the survey data are derived from adolescent self-report.

Approximately 11.3% (*n* = 505) of the initial study population were excluded for the logistic regression analysis due to missing data. Examination of differences between those with missing versus complete data was undertaken (available as an additional file (see Additional file 5)).

Residual confounding due to factors not included in this study remains possible.

Finally, generalisability to other high-income countries may be limited as the study is based upon adolescents living in Ireland with its unique sociocultural context.

## Conclusions

This study identified a high prevalence of ever binge drinking among 15–16-year-old adolescents in the West of Ireland, with nine risk factors and four protective factors independently associated with this alcohol use behaviour. There is a wide range of harms known to be associated with adolescent alcohol use, including injuries and poisonings, increased risks of other substance use, adverse effects on the developing brain and establishment of harmful patterns of consumption that may persist to adulthood. This represents an eminently preventable burden of ill-health and as such, this study supports an ongoing need for intersectoral action to protect adolescents from alcohol-related harm. Delaying use and denormalisation of alcohol use among adolescents must be a public health priority in Ireland and internationally. In the Irish context, the enactment of the Public Health (Alcohol) Act 2018—which aims to reduce alcohol-related harms to individuals, families, society and the economy—is the key policy development. This study highlights the need for continued implementation of all provisions of this legislation. The influence of parents, peers, and source of alcohol identified in this study highlights the importance of tailored and multi-modal public health messaging to communicate the risks of alcohol use to adolescents and their parents. Widespread parental awareness and understanding of the familial and wider social environmental factors associated with adolescent binge drinking is essential. Finally, this study also emphasises the need for further research on adolescent binge drinking in Ireland and internationally. Qualitative studies are particularly needed to explore in particular alcohol use culture specifically among Irish adolescents, and the relationship between adolescent team/club sports participation and alcohol use more broadly.

## Supplementary Information

**Additional file 1. ** Data Dictionary.

**Additional file 2.** Results of Univariable (Unadjusted) Logistic Regression.

**Additional file 3.** Analysis of Potential Risk and Protective Factors Associated with Ever Binge Drinking: Model 1 (Sociodemographic Factors).

**Additional file 4.** Analysis of Potential Risk and Protective Factors Associated with Ever Binge Drinking: Model 2 (Sociodemographic and Individual Factors) and Model 3 (Sociodemographic, Individual and Parent/Family Factors).

**Additional file 5.** Missing Data Analysis: Comparison of Participants with Complete versus Missing Data.

## Data Availability

The data that support the findings of this study are available from the Irish Social Science Data Archive (ISSDA) but restrictions apply to the availability of these data, which were used under license for the current study, and so are not publicly available. The ISSDA should be contacted for requests to access the Planet Youth Survey 2020 dataset (weblink: https://www.ucd.ie/issda/).

## Abbreviations

aOR: Adjusted odds ratio
CI: Confidence interval
ESPAD: European School Survey Project on Alcohol and Other Drugs
IQR: Interquartile range
OR: Odds ratio
PAC: Percentage accuracy in classification
SES: Socioeconomic status
Ref: Reference
WHO: World Health Organization

## References

[1] O’Dwyer C, Mongan D, Doyle A, Galvin B. HRB Overview Series 11 - Alcohol consumption, alcohol-related harm and alcohol policy in Ireland. Health Research Board. 2021. https://www.hrb.ie/fileadmin/2._Plugin_related_files/Publications/2021_publications/2021_HIE/Evidence_Centre/HRB_Alcohol_Overview_Series_11.pdf . Accessed 23 July 2022.

[2] World Health Organization. Global alcohol action plan 2022–2030 to strengthen implementation of the Global Strategy to Reduce the Harmful Use of Alcohol: First draft. 2021. https://www.drugsandalcohol.ie/34429/1/action-plan-on-alcohol_first-draft-final_formatted.pdf . Accessed 19 Sept 2022.

[3] World Health Organization. Alcohol. https://www.who.int/news-room/fact-sheets/detail/alcohol . Accessed 23 Jul 2022.

[4] Ritchie H, Rosner R. Alcohol Consumption. https://ourworldindata.org/alcohol-consumption#citation . Accessed 19 Sept 2022.

[5] The Organisation for Economic Co-Operation and Development. Health at a Glance 2021: OECD Indicators. 2021. https://www.oecd-ilibrary.org/docserver/ae3016b9-en.pdf?expires=1663617419&id=id&accname=guest&checksum=B7DC778B1E6D5DE1B3C800D7DA557CE1 . Accessed 19 Sept 2022.

[6] World Health Organization. Global Status Report on Alcohol and Health 2018. 2018. https://apps.who.int/iris/bitstream/handle/10665/274603/9789241565639-eng.pdf?sequence=1&isAllowed=y . Accessed 19 Sept 2022.

[7] World Health Organization – The Global Health Observatory. Harmful use of alcohol. https://www.who.int/data/gho/indicator-metadata-registry/imr-details/3415#:~:text=The%20strategy%20defines%20the%20harmful,of%20adverse%20health%20outcomes%20(hazardous . Accessed 19 Sept 2022.

[8] Department of Health. Healthy Ireland Survey Documents. https://www.gov.ie/en/collection/231c02-healthy-ireland-survey-wave/ . Accessed 23 July 2022.

[9] Mongan D, Millar SR, Galvin B. The 2019–20 Irish National Drug and Alcohol Survey: Main findings. Health Research Board. 2021. https://www.drugsandalcohol.ie/34287/1/HRB_Irish_National_Drug_and_Alcohol_Survey_2019_20.pdf . Accessed 23 July 2022.

[10] Degenhardt L, Stockings E, Patton G, Hall WD, Lynskey M (2016). The increasing global health priority of substance use in young people. Lancet Psychiatry.

[11] Alcohol Action Ireland. Alcohol Facts - Alcohol, children and young people. https://alcoholireland.ie/facts/childrenandyoungpeople/ . Accessed 23 July 2022.

[12] National Institute on Alcohol Abuse and Alcoholism. Alcohol Alert: Underage Drinking - Why Do Adolescents Drink, What Are the Risks, and How Can Underage Drinking Be Prevented? https://pubs.niaaa.nih.gov/publications/AA67/AA67.htm . Accessed 23 July 2022.

[13] The European School Survey Project on Alcohol and Other Drugs. Welcome to ESPAD. http://www.espad.org/ . Accessed 23 July 2022.

[14] Planet Youth. Planet Youth. https://planetyouth.org/ . Accessed 27 Sept 2022.

[15] Western Region Drug and Alcohol Taskforce/Planet Youth. Growing Up in the West. https://planetyouth.ie/ . Accessed 23 July 2022.

[16] National Health Service. Binge drinking. https://www.nhs.uk/live-well/alcohol-support/binge-drinking-effects/ . Accessed 19 Sept 2022.

[17] Centers for Disease Control and Prevention. Binge Drinking is a serious but preventable problem of excessive alcohol use. https://www.cdc.gov/alcohol/fact-sheets/bingedrinking.htm . Accessed 19 Sept 2022.

[18] The Centre for Addiction and Mental Health. Partying and getting drunk. https://www.camh.ca/en/health-info/guides-andpublications/partying-and-getting-drunk . Accessed 19 Sept 2022.

[19] Health Direct. Binge drinking. https://www.healthdirect.gov.au/binge-drinking . Accessed 19 Sept 2022.

[20] National Health and Medical Research Council. Alcohol. https://www.nhmrc.gov.au/health-advice/alcohol . Accessed 19 Sept 2022.

[21] Wellplace.nz. Defining problem drinking. https://wellplace.nz/facts-andinformation/alcohol/what-is-problem-drinking/ . Accessed 19 Sept 2022.

[22] Ball J, Edwards R, Sim D, Cook H, Denny S (2020). What explains the decline in adolescent binge-drinking in New Zealand?. Int J Drug Policy.

[23] Chen H-J, Balan S, Price RK (2012). Association of Contextual Factors with Drug Use and Binge Drinking among White Native American, and Mixed-Race Adolescents in the General Population. J Youth Adolesc.

[24] Diehl K, Thiel A, Zipfel S, Mayer J, Schneider S (2014). Substance use among elite adolescent athletes: Findings from the GOAL Study. Scand J of Med Sci Sports.

[25] Donath C, Gräßel E, Baier D, Pfeiffer C, Bleich S, Hillemacher T (2012). Predictors of binge drinking in adolescents: Ultimate and distal factors - A representative study. BMC Public Health.

[26] Grevenstein D, Nikendei C, Nagy E (2020). Alcohol Use, Binge Drinking, and Drunkenness Experience in Adolescence: Complex Associations with Family, Peers, Social Context, and Risk Perceptions. Subst Use Misuse.

[27] Guimarães MO, Paiva PC, Paiva HN, Lamounier JA, Ferreira EFE (2018). Religiosity as a possible protective factor against “binge drinking” among 12-year-old students: a population-based study. Cien Saude Colet..

[28] Hanewinkel R, Sargent JD, Hunt K, Sweeting H, Engels RCME, Scholte RHJ (2014). Portrayal of Alcohol Consumption in Movies and Drinking Initiation in Low-Risk Adolescents. Pediatrics.

[29] Hodder RK, Campbell E, Gilligan C, Lee H, Lecathelinais C, Green S (2018). Association between Australian adolescent alcohol use and alcohol use risk and protective factors in 2011 and 2014. Drug Alcohol Rev.

[30] Huang R, Ho SY, Wang MP, Lo WS, Lam TH (2016). Sociodemographic risk factors of alcohol drinking in Hong Kong adolescents. J Epidemiol Community Health.

[31] Jonkman H, Steketee M, Tombourou JW, Cini K, Williams J (2014). Community variation in adolescent alcohol use in Australia and the Netherlands. Health Promot Int.

[32] Maggs JL, Staff J, Patrick ME, Wray-Lake L, Schulenberg JE (2015). Alcohol use at the cusp of adolescence: A prospective national birth cohort study of prevalence and risk factors. J Adolesc Health.

[33] Patrick ME, Evans-Polce R, Terry-McElrath YM (2019). Faster Escalation from First Drink to First Intoxication as a Risk Factor for Binge and High-intensity Drinking among Adolescents. Addict Behav.

[34] Mu KJ, Moore SE, LeWinn KZ (2015). Internet use and adolescent binge drinking: Findings from the Monitoring the Future study. Addict Behav Rep.

[35] Stickley A, Koyanagi A, Koposov R, McKee M, Roberts B, Murphy A (2013). Binge drinking among adolescents in Russia: Prevalence, risk and protective factors. Addict Behav.

[36] Patte KA, Leatherdale ST (2017). A cross-sectional analysis examining the association between dieting behaviours and alcohol use among secondary school students in the COMPASS study. J Public Health (Oxf).

[37] Pedersen W, von Soest T (2015). Adolescent alcohol use and binge drinking: An 18-year trend study of prevalence and correlates. Alcohol.

[38] Stoolmiller M, Wills TA, McClure AC, Tanski SE, Worth KA, Gerrard M (2012). Comparing media and family predictors of alcohol use: A cohort study of US adolescents. BMJ Open.

[39] Zarzar PM, Jorge KO, Oksanen T, Vale MP, Ferreira EF, Kawachi I (2012). Association between binge drinking, type of friends and gender: A cross-sectional study among Brazilian adolescents. BMC Public Health.

[40] Butler A, Romano I, Patte K, Ferro MA, de Groh M, Jiang Y (2019). Psychological correlates and binge drinking behaviours among Canadian youth: A cross-sectional analysis of the mental health pilot data from the COMPASS study. BMJ Open.

[41] Elisaus P, Williams G, Bourke M, Clough G, Harrison A, Verma A (2018). Factors associated with the prevalence of adolescent binge drinking in the urban areas of Greater Manchester. Eur J Public Health.

[42] Holligan SD, Qian W, de Groh M, Jiang Y, Leatherdale ST (2020). Micro-level factors associated with alcohol use and binge drinking among youth in the COMPASS study (2012/13 to 2017/18). Health Promot Chronic Dis Prev Can.

[43] Holligan SD, Battista K, de Groh M, Jiang Y, Leatherdale ST (2019). Age at first alcohol use predicts current alcohol use, binge drinking and mixing of alcohol with energy drinks among Ontario Grade 12 students in the COMPASS study. Health Promot Chronic Dis Prev Can.

[44] Jackson N, Denny S, Sheridan J, Zhao J, Ameratunga S (2016). The role of neighborhood disadvantage, physical disorder, and collective efficacy in adolescent alcohol use: A multilevel path analysis. Health Place.

[45] Jorge KO, Ferreira RC, Ferreira EFE, Vale MP, Kawachi I, Zarzar PM (2017). Binge drinking and associated factors among adolescents in a city in southeastern Brazil: A longitudinal study. Cad Saude Publica.

[46] Kristjansson AL, Lilly CL, Thorisdottir IE, Allegrante JP, Mann MJ, Sigfusson J (2021). Testing risk and protective factor assumptions in the Icelandic model of adolescent substance use prevention. Health Educ Res.

[47] Kristjansson AL, James JE, Allegrante JP, Sigfusdottir ID, Helgason AR (2010). Adolescent substance use, parental monitoring, and leisure-time activities: 12-year outcomes of primary prevention in Iceland. Prev Med.

[48] Haase T, Pratschke J. Risk and protection factors for substance use among young people: a comparative study of early school-leavers and school-attending students. The Stationery Office. 2010. https://www.drugsandalcohol.ie/14100/1/NACD_RiskYoungPeopleSchool.pdf . Accessed 23 July 2022.

[49] Department of Health. Ireland placed on Level 3 of the Plan for Living with COVID19. https://www.gov.ie/en/press-release/5b068-irelandplaced-on-level-3-of-the-plan-for-living-with-covid-with-special-measures-for-asafe-christmas/ . Accessed 19 Sept 2022.

[50] Department of Health. Ireland placed on Level 5 Restrictions of the Plan for Living with COVID-19 - with a number of specific adjustments. https://www.gov.ie/en/press-release/a1f21-ireland-placed-onlevel-5-restrictions-of-the-plan-for-living-with-covid-19-with-a-number-of-specificadjustments/ . Accessed 19 Sept 2022.

[51] Staff J, Maggs JL (2020). Parents allowing drinking is associated with adolescents’ heavy alcohol use. Alcohol Clin Exp Res.

[52] Terry-McElrath YM, Stern SA, Patrick ME (2017). Do alcohol use reasons and contexts differentiate adolescent high-intensity drinking? Data from U.S. high school seniors, 2005-2016. Data from. Psychol Addict Behav..

[53] Rossheim ME, Stephenson CJ, Thombs DL, Livingston MD, Walters ST, Suzuki S (2017). Characteristics of drinking events associated with heavy episodic drinking among adolescents in the United States. Drug Alcohol Depend.

[54] Rowan ZR (2016). Social Risk Factors of Black and White Adolescents’ Substance Use: The Differential Role of Siblings and Best Friends. J Youth Adolesc.

[55] Kwok KHR, Yuan SNV (2016). Parental socioeconomic status and binge drinking in adolescents: A systematic review. Am J Addict.

[56] Danielsson AK, Romelsjö A, Tengström A (2011). Heavy episodic drinking in early adolescence: Gender-specific risk and protective factors. Subst Use Misuse.

[57] Halladay J, Woock R, El-Khechen H, Munn C, MacKillop J, Amlung M (2020). Patterns of substance use among adolescents: a systematic review. Drug Alcohol Depend.

[58] Yap MBH, Cheong TWK, Zaravinos-Tsakos F, Lubman DI, Jorm AF (2017). Modifiable parenting factors associated with adolescent alcohol misuse: A systematic review and meta-analysis of longitudinal studies. Addiction.

[59] Zuquetto CR, Opaleye ES, Feijó MR, Amato TC, Ferri CP, Noto AR (2019). Contributions of parenting styles and parental drunkenness to adolescent drinking. Braz J Psychiatry.

[60] Patrick ME, Schulenberg JE (2013). Prevalence and Predictors of Adolescent Alcohol Use and Binge Drinking in the United States. Alcohol Res.

[61] Martins-Oliveira JG, Kawachi I, Paiva PCP, de Paiva HN, Pordeus IA, Zarzar PM (2018). Correlates of binge drinking among Brazilian adolescents. Cien Saude Colet.

[62] Marshall EJ (2014). Adolescent alcohol use: Risks and consequences. Alcohol.

[63] Siqueira L, Smith VC (2015). Binge drinking. Pediatrics..

[64] Chung SS, Joung KH (2019). Risk Factors Related to Binge Drinking: A comparative study of American and South Korean adolescents. J Sch Nurs.

[65] Mattick RP, Wadolowski M, Aiken A, Clare PJ, Hutchinson D, Najman J (2017). Parental supply of alcohol and alcohol consumption in adolescence: Prospective cohort study. Psychol Med.

