# Supplementary material for: Adolescent binge drinking in the West of Ireland: associated risk and protective factors
Source: BMC Public Health. 2023 Jun 5;23:1064. doi: 10.1186/s12889-023-15577-z (PMC10240125; doi:10.1186/s12889-023-15577-z)
Supplement: Supplementary file 1 — Additional file 1. Data Dictionary. [file 12889_2023_15577_MOESM1_ESM.docx]

**Additional File 1: Data Dictionary.**

| **Full Name of Variable** | **Source** | **Question Within Planet Youth Questionnaire** | **Type of Variable** | **Original Coding** | **Recoding Completed** |
| --- | --- | --- | --- | --- | --- |
| Binge drinking | Derived | How often have you had:   - 5 (five) or more alcoholic drinks within a two-hour period or less? | Categorical | 1 = Never, 2 = 1-2 times, 3 = 3-5 times, 4 = 6-9 times, 5 = 10-19 times, 6 = 20-39 times, 7 = 40 times or more. | 0 = Never (1)  1 = Ever (2-7) |
| Gender | Derived | How would you describe your gender? | Categorical | 1 = Male, 2 = Female, 3 = Non-binary, 4 = Prefer not to say | 0 = Male (1)  1 = Female (2)  3 and 4 recoded with missing variables due to insufficient numbers for inclusion in analysis. |
| Ethnicity | Derived | What is your ethnic/cultural background? | Categorical | 1 = Irish, 2 = Irish Traveller, 3 = Any other White background, 4 = Asian Irish, 5 = Chinese, 6 = Any other Asian background, 7 = Black Irish, 8 = African, 9 = Any other background, 10 = Roma, 11 = Other | 0 = White background (1-3)  1 = Non-White background (4-11) |

| **Full Name of Variable** | **Source** | **Question Within PY Questionnaire** | **Type of Variable** | **Original Coding** | **Recoding Completed** |
| --- | --- | --- | --- | --- | --- |
| Maternal education | Derived | What is the highest level of education your mother/carer completed? | Categorical | 1 = Postgraduate Degree (Masters or Doctorate), 2 = Degree from University, 3 = Diploma from a Technical Institute, 4 = Completed Leaving Certificate, 5 = Completed Junior Certificate, 6 = Completed Primary School or less, 7 = Don’t Know | 0 = Tertiary (1-3)  1 = Secondary (4)  2 = Primary (5-6)  3 = Didn’t Know (7) |
| Self-rated mental health | Derived | How would you rate your mental health? | Categorical | 1 = Very Good, 2 = Good, 3 = Okay, 4 = Bad, 5 = Very Bad | 0 = Very good/good (1,2)  1 = Okay (3)  2 = Bad/very bad (4-5) |
| Current cigarette use | Derived | How many cigarettes, on average, have you smoked in the last 30 days? | Categorical | 1 = None, 2 = Less than one a week, 3 = Less than one a day, 4 = 1-5 a day, 5 = 6-10 a day, 6 = 11-20 a day, 7 = More than 20 a day | 0 = Non-current user (1)  1 = Current smoker (2-7) |
| Current cannabis use | Derived | How often have you used cannabis substances?  During the last 30 days | Categorical | 1 = None, 2 = 1-2 times, 3 = 3-5 times, 4 = 6-9 times, 5 = 10-19 times, 6 = 20-39 times, 7 = 40 times or more | 0 = Non-current user (1)  1 = Current user (2-7) |

| **Full Name of Variable** | **Source** | **Question Within PY Questionnaire** | **Type of Variable** | **Original Coding** | **Recoding Completed** |
| --- | --- | --- | --- | --- | --- |
| Parental supervision | Derived | How do the following statements apply to you?   - My parents/carers know who I am with in the evenings - My parents/carers know where I am in the evenings | Scale  /Continuous | 1 = Very Well, 2 = Well, 3 = Poorly, 4 = Very poorly | 1 = Very Poorly (4)  2 = Poorly (3)  3 = Well (2)  4 = Very well (1)  Scale 2-8 |
| Parental drunkenness | Derived | Do any of the following people become drunk at least once each week?   - Father - Mother | Categorical | 1 = No, 2 = Yes, 3 = Doesn’t Apply | 0 = No (1, 3)  1 = Yes (2)  Either parent response as Yes was taken as Yes |
| Perceived parental reaction to drunkenness | Derived | How do you think your parents/carers would react if you did any of the following?   - Got drunk | Categorical | 1 = Totally against it, 2 = Against it, 3 = A bit against it, 4 = They would not care | 0 = A bit against it/they would not care (3,4)  1 = Totally against it/against it (1,2) |
| Gets alcohol from parent/carer | Derived | How do you usually get the alcohol you drink?   - My parent/carer gives it to me | Categorical | 1 = Never, 2 = Rarely, 3 = Sometimes, 4 = Often, 5 = Almost always | 0 = Never/Rarely (1-2)  1 = Sometimes/Often/Always (3-5) |
| Having friends that drink alcohol | Derived | How many of your friends do you think do the following?   - Drink alcohol | Categorical | 1 = None, 2 = A few, 3 = Some, 4 = Most, 5 = Almost all | 0 = None (1)  1 = Any (2-5) |

| **Full Name of Variable** | **Source** | **Question Within PY Questionnaire** | **Type of Variable** | **Original Coding** | **Recoding Completed** |
| --- | --- | --- | --- | --- | --- |
| School engagement |  | How well do the following statements apply to you?   - I find schoolwork pointless - I find schoolwork boring - I am poorly prepared for classes - I feel I don’t put enough effort into my schoolwork | Scale  /Continuous | 1 = Almost always, 2 = Often, 3 = Sometimes, 4 = Rarely, 5 = Almost never | 1 = Almost always  2 = Often  3 = Sometimes  4 = Rarely  5 = Almost never  Scale 4-20 |
| Team/club sports participation | Derived | How many times a week do you?   - Engage in sports outside school with a club or team? | Categorical | 1 = Never, 2 = Once a week, 3 = Twice a week, 4 = 3 times a week, 5 = 4 times a week, 6 = 5 times a week, 7 = 6 times a week, 8 = Every day | 0 = Never (1)  1 = 1-4 times/week (2-5)  2 = 5 or more times/week (6-8) |
| Gets alcohol from friends | Derived | How do you usually get the alcohol you drink?   - I get it from friends or schoolmates | Categorical | 1 = Never, 2 = Rarely, 3 = Sometimes, 4 = Often, 5 = Almost always | 0 = Never/Rarely (1-2)  1 = Sometimes/Often/Always (3-5) |
| Gets alcohol from another adult | Derived | How do you usually get the alcohol you drink?   - Another adult gets it for me | Categorical | 1 = Never, 2 = Rarely, 3 = Sometimes, 4 = Often, 5 = Almost always | 0 = Never/Rarely (1-2)  1 = Sometimes/Often/Always (3-5) |
