# Supplementary material for: Adolescent binge drinking in the West of Ireland: associated risk and protective factors
Source: BMC Public Health. 2023 Jun 5;23:1064. doi: 10.1186/s12889-023-15577-z (PMC10240125; doi:10.1186/s12889-023-15577-z)
Supplement: Supplementary file 2 — Additional file 2. Results of Univariable (Unadjusted) Logistic Regression. [file 12889_2023_15577_MOESM2_ESM.docx]

**Additional File 2: Results of Univariable (Unadjusted) Logistic Regression.**

|  | **Binge Drinking (Ever vs. Never)** | | |
| --- | --- | --- | --- |
| **Variables** | **OR** | **95% CI** | **p-value** |
| **Sociodemographic** |  |  |  |
| Gender |  |  |  |
| Male | Ref |  |  |
| Female | 0.88 | 0.77-1.00 | 0.05 |
| Ethnicity |  |  |  |
| White | Ref |  |  |
| Non-White | 0.52 | 0.38-0.71 | <0.001 |
| Maternal Education |  |  |  |
| Tertiary | Ref |  |  |
| Secondary | 1.23 | 1.05-1.45 | 0.012 |
| Primary | 1.22 | 0.90-1.65 | 0.197 |
| Didn’t Know | 0.97 | 0.81-1.16 | 0.707 |
| **Individual** |  |  |  |
| Mental Health |  |  |  |
| Very good/good | Ref |  |  |
| Okay | 1.29 | 1.11-1.51 | 0.001 |
| Bad/very bad | 2.24 | 1.89-2.67 | <0.001 |
| Current cigarette use |  |  |  |
| No | Ref |  |  |
| Yes | 10.69 | 8.44-13.54 | <0.001 |
| Current cannabis use |  |  |  |
| No | Ref |  |  |
| Yes | 12.75 | 8.96-18.13 | <0.001 |
| **Parents and family** |  |  |  |
| Parental supervision |  |  |  |
| 1 SD increase corresponds to | 0.56 | 0.52-0.60 | <0.001 |
| Parental drunkenness |  |  |  |
| No/less than weekly | Ref |  |  |
| At least weekly | 2.39 | 2.01-2.84 | <0.001 |
| Parental reaction to drunkenness |  |  |  |
| A bit against/wouldn’t care | Ref |  |  |
| Totally against/against it | 0.28 | 0.25-0.32 | <0.001 |
| Gets alcohol from parents |  |  |  |
| Never/rarely | Ref |  |  |
| Sometimes/often/almost always | 2.94 | 2.49-3.48 | <0.001 |
| **Peer Group** |  |  |  |
| Having friends that drink alcohol |  |  |  |
| No | Ref |  |  |
| Yes | 18.44 | 10.97-30.98 | <0.001 |

*Appendix Continued*

|  | **Binge Drinking (Ever vs. Never)** | | |
| --- | --- | --- | --- |
| **Variables** | **OR** | **95% CI** | **p-value** |
| **School** |  |  |  |
| School engagement |  |  |  |
| 1 SD increase corresponds to | 1.29 | 1.20-1.38 | <0.001 |
| **Leisure Time/Source of Alcohol in Local Community** |  |  |  |
| Team/club sports participation |  |  |  |
| Never | Ref |  |  |
| 1-4 times/week | 1.20 | 1.05-1.38 | 0.009 |
| 5 or more times/week | 1.38 | 1.06-1.79 | 0.017 |
| Gets alcohol from friends |  |  |  |
| Never/rarely | Ref |  |  |
| Sometimes/often/almost always | 10.03 | 8.61-11.70 | <0.001 |
| Gets alcohol from another adult |  |  |  |
| Never/rarely | Ref |  |  |
| Sometimes/often/almost always | 11.59 | 9.63-13.96 | <0.001 |

OR = Odds Ratio; 95% CI = 95% Confidence Interval; Ref = Reference Group; SD = Standard Deviation.
