# Supplementary material for: Adolescent binge drinking in the West of Ireland: associated risk and protective factors
Source: BMC Public Health. 2023 Jun 5;23:1064. doi: 10.1186/s12889-023-15577-z (PMC10240125; doi:10.1186/s12889-023-15577-z)
Supplement: Supplementary file 3 — Additional file 3. Analysis of Potential Risk and Protective Factors Associated with Ever Binge Drinking: Model 1 (Sociodemographic Factors). [file 12889_2023_15577_MOESM3_ESM.docx]

**Additional File 3: Analysis of Potential Risk and Protective Factors Associated with Ever Binge Drinking: Model 1 (Sociodemographic Factors).**

|  | **Binge Drinking (Ever vs. Never)** | | |
| --- | --- | --- | --- |
|  | **Model 1 (n=4262)** | | |
| **Variables** | **aOR** | **95% CI** | **p-value** |
| Gender |  |  |  |
| Male | Ref |  |  |
| Female | 0.86 | 0.76-0.98 | 0.021 |
| Ethnicity |  |  |  |
| White | Ref |  |  |
| Non-White | 0.56 | 0.42-0.75 | <0.001 |
| Maternal Education |  |  |  |
| Tertiary | Ref |  |  |
| Secondary | 1.22 | 1.04-1.43 | 0.014 |
| Primary | 1.22 | 0.91-1.63 | 0.179 |
| Didn’t Know | 0.92 | 0.78-1.10 | 0.362 |

aOR = Adjusted Odds Ratio; 95% CI = 95% Confidence Interval; Ref = Reference Group. Model 1: Nagelkerke r^2^ = 0.011; Percentage Accuracy in Classification: 65.9%
