# Supplementary material for: Adolescent binge drinking in the West of Ireland: associated risk and protective factors
Source: BMC Public Health. 2023 Jun 5;23:1064. doi: 10.1186/s12889-023-15577-z (PMC10240125; doi:10.1186/s12889-023-15577-z)
Supplement: Supplementary file 4 — Additional file 4. Analysis of Potential Risk and Protective Factors Associated with Ever Binge Drinking: Model 2 (Sociodemographic and Individual Factors) and Model 3 (Sociodemographic, Individual and Parent/Family Factors). [file 12889_2023_15577_MOESM4_ESM.docx]

**Additional File 4: Analysis of Potential Risk and Protective Factors Associated with Ever Binge Drinking: Model 2 (Sociodemographic and Individual Factors) and Model 3 (Sociodemographic, Individual and Parent/Family Factors).**

|  | **Binge Drinking (Ever vs. Never)** | | | | | |
| --- | --- | --- | --- | --- | --- | --- |
|  | **Model 2 (n=4205)** | | | **Model 3 (n=4131)** | | |
| **Variables** | **aOR** | **95% CI** | **p-value** | **aOR** | **95% CI** | **p-value** |
| **Sociodemographic** |  |  |  |  |  |  |
| Gender |  |  |  |  |  |  |
| Male | Ref |  |  | Ref |  |  |
| Female | 0.83 | 0.72-0.96 | 0.010 | 0.90 | 0.77-1.05 | 0.164 |
| Ethnicity |  |  |  |  |  |  |
| White | Ref |  |  | Ref |  |  |
| Non-White | 0.45 | 0.32-0.63 | <0.001 | 0.45 | 0.31-0.65 | <0.001 |
| Maternal Education |  |  |  |  |  |  |
| Tertiary | Ref |  |  | Ref |  |  |
| Secondary | 1.15 | 0.96-1.36 | 0.123 | 1.05 | 0.88-1.27 | 0.580 |
| Primary | 1.15 | 0.83-1.57 | 0.404 | 1.01 | 0.72-1.43 | 0.936 |
| Didn’t Know | 0.85 | 0.70-1.03 | 0.103 | 0.82 | 0.67-1.00 | 0.053 |
| **Individual** |  |  |  |  |  |  |
| Mental Health |  |  |  |  |  |  |
| Very good/good | Ref |  |  | Ref |  |  |
| Okay | 1.18 | 1.00-1.38 | 0.050 | 1.00 | 0.85-1.19 | 0.964 |
| Bad/very bad | 1.72 | 1.43-2.08 | <0.001 | 1.40 | 1.14-1.72 | 0.002 |
| Current cigarette use |  |  |  |  |  |  |
| No | Ref |  |  | Ref |  |  |
| Yes | 6.95 | 5.47-8.85 | <0.001 | 5.49 | 4.25-7.10 | <0.001 |
| Current cannabis use |  |  |  |  |  |  |
| No | Ref |  |  | Ref |  |  |
| Yes | 5.74 | 3.92-8.42 | <0.001 | 4.27 | 2.85-6.39 | <0.001 |
| **Parents and family** |  |  |  |  |  |  |
| Parental supervision |  |  |  |  |  |  |
| 1 SD increase corresponds to |  |  |  | 0.64 | 0.59-0.69 | <0.001 |
| Parental drunkenness |  |  |  |  |  |  |
| No/less than weekly |  |  |  | Ref |  |  |
| At least weekly |  |  |  | 1.26 | 1.02-1.54 | 0.030 |
| Parental reaction to drunkenness |  |  |  |  |  |  |
| A bit against/  wouldn’t care |  |  |  | Ref |  |  |
| Totally against/  against it |  |  |  | 0.40 | 0.34-0.47 | <0.001 |

*Table Continued*

|  | **Ever Binge Drinking vs. Never** | | | | | |
| --- | --- | --- | --- | --- | --- | --- |
|  | **Model 2 (n=4205)** | | | **Model 3 (n=4131)** | | |
| **Variables** | **aOR** | **95% CI** | **p-value** | **aOR** | **95% CI** | **p-value** |
| Gets alcohol from parents |  |  |  |  |  |  |
| Never/rarely |  |  |  | Ref |  |  |
| Sometimes/often/  almost always |  |  |  | 1.86 | 1.53-2.26 | <0.001 |

aOR = Adjusted Odds Ratio. 95% CI = 95% Confidence Interval. Ref = Reference Group; SD = Standard Deviation. Model 2: Nagelkerke r^2^ = 0.212; Percentage Accuracy in Classification: 74.6%. Model 3: Nagelkerke r^2^ = 0.318; Percentage Accuracy in Classification: 75.8%

**Supplementary File 4: Analysis of Potential Risk and Protective Factors Associated with Ever Binge Drinking: Model 4 (Sociodemographic, Individual, Parent/Family and Peer Group Factors) and Model 5 (Sociodemographic, Individual, Parent/Family, Peer Group and School Factors).**

|  | **Binge Drinking (Ever vs. Never)** | | | | | |
| --- | --- | --- | --- | --- | --- | --- |
|  | **Model 4 (n=4118)** | | | **Model 5 (n=4014)** | | |
| **Variables** | **aOR** | **95% CI** | **p-value** | **aOR** | **95% CI** | **p-value** |
| **Sociodemographic** |  |  |  |  |  |  |
| Gender |  |  |  |  |  |  |
| Male | Ref |  |  | Ref |  |  |
| Female | 0.86 | 0.73-1.01 | 0.060 | 0.87 | 0.74-1.03 | 0.097 |
| Ethnicity |  |  |  |  |  |  |
| White | Ref |  |  | Ref |  |  |
| Non-White | 0.46 | 0.32-0.67 | <0.001 | 0.48 | 0.33-0.70 | <0.001 |
| Maternal Education |  |  |  |  |  |  |
| Tertiary | Ref |  |  | Ref |  |  |
| Secondary | 1.09 | 0.90-1.31 | 0.382 | 1.04 | 0.86-1.26 | 0.691 |
| Primary | 1.03 | 0.73-1.47 | 0.858 | 1.03 | 0.72-1.47 | 0.888 |
| Didn’t Know | 0.90 | 0.73-1.11 | 0.306 | 0.88 | 0.71-1.09 | 0.250 |
| **Individual** |  |  |  |  |  |  |
| Mental Health |  |  |  |  |  |  |
| Very good/good | Ref |  |  | Ref |  |  |
| Okay | 0.98 | 0.83-1.17 | 0.860 | 0.98 | 0.82-1.17 | 0.802 |
| Bad/very bad | 1.42 | 1.15-1.75 | 0.001 | 1.43 | 1.15-1.77 | 0.001 |
| Current cigarette use |  |  |  |  |  |  |
| No | Ref |  |  | Ref |  |  |
| Yes | 5.07 | 3.91-6.57 | <0.001 | 5.13 | 3.94-6.68 | <0.001 |
| Current cannabis use |  |  |  |  |  |  |
| No | Ref |  |  | Ref |  |  |
| Yes | 4.04 | 2.70-6.06 | <0.001 | 3.98 | 2.65-5.98 | <0.001 |
| **Parents and family** |  |  |  |  |  |  |
| Parental supervision |  |  |  |  |  |  |
| 1 SD increase corresponds to | 0.66 | 0.61-0.72 | <0.001 | 0.67 | 0.61-0.72 | <0.001 |
| Parental drunkenness |  |  |  |  |  |  |
| No/less than weekly | Ref |  |  | Ref |  |  |
| At least weekly | 1.24 | 1.01-1.53 | 0.039 | 1.25 | 1.01-1.54 | 0.037 |
| Parental reaction to drunkenness |  |  |  |  |  |  |
| A bit against/  wouldn’t care | Ref |  |  | Ref |  |  |
| Totally against/  against it | 0.44 | 0.38-0.52 | <0.001 | 0.44 | 0.37-0.51 | <0.001 |

*Table Continued*

|  | **Binge Drinking (Ever vs. Never)** | | | | | |
| --- | --- | --- | --- | --- | --- | --- |
|  | **Model 4 (n=4118)** | | | **Model 5 (n=4014)** | | |
| **Variables** | **aOR** | **95% CI** | **p-value** | **aOR** | **95% CI** | **p-value** |
| Gets alcohol from parents |  |  |  |  |  |  |
| Never/rarely | Ref |  |  | Ref |  |  |
| Sometimes/often/  almost always | 1.75 | 1.44-2.13 | <0.001 | 1.72 | 1.41-2.10 | <0.001 |
| **Peer Group** |  |  |  |  |  |  |
| Having friends that drink alcohol |  |  |  |  |  |  |
| No | Ref |  |  |  |  |  |
| Yes | 9.38 | 5.61-15.69 | <0.001 | 9.30 | 5.55-15.59 | <0.001 |
| **School** |  |  |  |  |  |  |
| School engagement |  |  |  |  |  |  |
| 1 SD increase corresponds to |  |  |  | 1.22 | 1.13-1.32 | <0.001 |

aOR = Adjusted Odds Ratio. 95% CI = 95% Confidence Interval. Ref = Reference Group; SD = Standard Deviation. Model 4: Nagelkerke r^2^ = 0.352; Percentage Accuracy in Classification: 75.9% Model 5: Nagelkerke r^2^ = 0.359; Percentage Accuracy in Classification: 76.0%
