# Supplementary material for: Adolescent binge drinking in the West of Ireland: associated risk and protective factors
Source: BMC Public Health. 2023 Jun 5;23:1064. doi: 10.1186/s12889-023-15577-z (PMC10240125; doi:10.1186/s12889-023-15577-z)
Supplement: Supplementary file 5 — Additional file 5. Missing Data Analysis: Comparison of Participants with Complete versus Missing Data. [file 12889_2023_15577_MOESM5_ESM.docx]

**Additional File 5: Missing Data Analysis – Comparison of Participants with Complete versus Missing Data.**

|  | **Valid** | **Participants with complete data**  **(n = 3968)** | **Participants excluded due to missing data**  **(n = 505)** |  |
| --- | --- | --- | --- | --- |
| **Variables** | **n** | **n (%)** | **n (%)** | **p-value** |
| **Binge Drinking Status** | 4390 |  |  |  |
| Ever |  | 1344 (36.3) | 153 (33.9) |  |
| Never |  | 2624 (63.7) | 269 (66.1) | 0.353 |
| **Sociodemographic** |  |  |  |  |
| Gender | 4395 |  |  |  |
| Male |  | 1936 (48.8) | 236 (55.3) |  |
| Female |  | 2032 (51.2) | 191 (44.7) | 0.013 |
| Ethnicity | 4454 |  |  |  |
| White |  | 3724 (93.9) | 438 (90.1) |  |
| Non-White |  | 244 (6.1) | 48 (9.9) | 0.002 |
| Maternal Education | 4429 |  |  |  |
| Tertiary |  | 2110 (53.2) | 210 (45.5) |  |
| Secondary |  | 914 (23.0) | 80 (17.4) |  |
| Primary |  | 196 (4.9) | 34 (7.4) |  |
| Didn’t Know |  | 748 (18.9) | 137 (29.7) | <0.001 |
| **Individual** |  |  |  |  |
| Mental Health | 4446 |  |  |  |
| Very good/good |  | 1897 (47.8) | 204 (42.7) |  |
| Okay |  | 1294 (32.6) | 153 (32.0) |  |
| Bad/very bad |  | 777 (19.6) | 121 (25.3) | 0.009 |
| Current cigarette use | 4434 |  |  |  |
| Yes |  | 480 (12.1) | 80 (17.2) |  |
| No |  | 3488 (87.9) | 386 (82.8) | 0.002 |
| Current cannabis use | 4395 |  |  |  |
| Yes |  | 250 (6.3) | 41 (9.6) |  |
| No |  | 3718 (93.7) | 386 (90.4) | 0.012 |
| **Parents and family** |  |  |  |  |
| Parental supervision | 4419 |  |  |  |
| Median (IQR) |  | 7 (2) | 7 (2) | 0.189 |
| Parental drunkenness | 4432 |  |  |  |
| At least weekly |  | 3338 (84.1) | 390 (84.1) |  |
| No/less than weekly |  | 630 (15.9) | 74 (15.9) | 1.000 |
| Parental reaction to drunkenness | 4399 |  |  |  |
| A bit against/wouldn’t care |  | 1522 (38.4) | 170 (39.4) |  |
| Totally against/against it |  | 2446 (61.6) | 261 (60.6) | 0.698 |

*Table Continued*

|  | **Valid** | **Participants with complete data**  **(n = 3968)** | **Participants excluded due to missing data**  **(n = 505)** |  |
| --- | --- | --- | --- | --- |
| **Variables** | **n** | **n (%)** | **n (%)** | **p-value** |
| Gets alcohol from parents | 4396 |  |  |  |
| Never/rarely |  | 3264 (82.3) | 341 (79.7) |  |
| Sometimes/often/almost always |  | 704 (17.7) | 87 (20.3) | 0.209 |
| **Peer Group** |  |  |  |  |
| Having friends that drink alcohol | 4395 |  |  |  |
| Yes |  | 3501 (88.2) | 371 (86.9) |  |
| No |  | 467 (11.8) | 56 (13.1) | 0.461 |
| **School** |  |  |  |  |
| School engagement | 4353 |  |  |  |
| Mean (sd) |  | 12.8 (2.9) | 12.8 (3.1) | 0.790 |
| **Leisure Time/Source of Alcohol in Local Community** |  |  |  |  |
| Team/club sports participation | 4435 |  |  |  |
| Never |  | 1777 (44.8) | 237 (50.8) |  |
| 1-4 times/week |  | 1914 (48.2) | 199 (42.6) |  |
| 5 or more times/week |  | 277 (7.0) | 31 (6.6) | 0.047 |
| Gets alcohol from friends | 4387 |  |  |  |
| Never/rarely |  | 2660 (67.0) | 268 (64.0) |  |
| Sometimes/often/almost always |  | 1308 (33.0) | 151 (36.0) | 0.224 |
| Gets alcohol from another adult | 4392 |  |  |  |
| Never/rarely |  | 3169 (79.9) | 331 (78.1) |  |
| Sometimes/often/almost always |  | 799 (20.1) | 93 (21.9) | 0.417 |

IQR = Interquartile Range; sd = Standard Deviation.
